# Modified Descemet's Stripping Automated Endothelial Keratoplasty: The Use of Ophthalmic Viscoelastic Devices in Hypotonic Eyes That Had Undergone Glaucoma Filtering Surgeries

**DOI:** 10.1155/2018/9387810

**Published:** 2018-02-01

**Authors:** Itaru Oyakawa, Takahiko Hayashi, Toshiki Shimizu, Naoko Kato

**Affiliations:** ^1^Department of Ophthalmology, Heart Life Hospital, Okinawa, Japan; ^2^Department of Ophthalmology, Ryukyu University, Okinawa, Japan; ^3^Department of Ophthalmology, Yokohama Minami Kyosai Hospital, Kanagawa, Japan; ^4^Department of Ophthalmology, Saitama Medical University, Saitama, Japan

## Abstract

**Purpose:**

Descemet's stripping automated endothelial keratoplasty (DSAEK) is more difficult in hypotonic eyes with filtering bleb, due to the difficulties in elevating the intraocular pressure (IOP). We report a new method that uses ophthalmic viscoelastic devices (OVDs) to achieve good graft adhesion.

**Case Presentation:**

We performed modified DSAEK surgery on 2 eyes of 2 patients, who had previously undergone a trabeculectomy. Both eyes had functioning filtering blebs; the IOP was lower than 10 mmHg without medication. After the graft was inserted into the anterior chamber, the conjunctiva was penetrated, apart from the bleb, using a 30 G needle, and Healon V® was injected into the bleb until the encapsulated space was filled completely. Air was subsequently injected into the anterior chamber to promote the graft attachment to the back surface of the cornea. The IOP was elevated above 40 mmHg in both eyes 1 h after surgery and then decreased to less than 30 mmHg over the subsequent 3 h period. The implanted graft showed good adhesion and no dislocation.

**Conclusions:**

Our novel DSAEK procedure that adds one step of OVD injection into the filtering bleb may be useful for hypotonic eyes that had undergone filtering surgeries.

## 1. Introduction

Descemet's stripping automated endothelial keratoplasty (DSAEK) has recently become the most popular surgical treatment for corneal endothelial dysfunction worldwide [1, 2]. With an increasing number of DSAEK surgeries, ophthalmologists are more likely to encounter eyes with various preexisting ocular diseases. For example, DSAEK is more challenging in eyes that have previously undergone glaucoma filtering surgeries [3–7]. Alternate outflow pathways created by filtering surgeries may lead to difficulties obtaining optimal air filling in the anterior chamber, complicating the attachment of the implanted graft. It is important to maintain a sufficiently high intraocular pressure (IOP) during and shortly after surgery, to promote the attachment of the implanted graft. Banitt et al. [8] reported using an overfilling technique, and Liang et al. [9] used a continuous air-pumping method to obtain a sufficiently high IOP during DSAEK surgery. However, these methods are limited in that the implanted graft may become detached or dislocated if there is air movement from the anterior chamber to the filtering bleb after surgery. We developed a novel method that involves the injection of ophthalmic viscoelastic devices (OVDs) into functioning blebs during DSAEK surgery. Here, we describe two cases with hypotonic eyes after successful filtering surgery for glaucoma that underwent endothelial keratoplasty using our modified DSAEK procedure.

## 2. Case Presentation

We used the DSAEK surgical technique in this case report. This technical report followed the tenets of the Declaration of Helsinki and all patients signed an informed consent form before DSAEK.

**Case 1 .**

**Figure 1:**
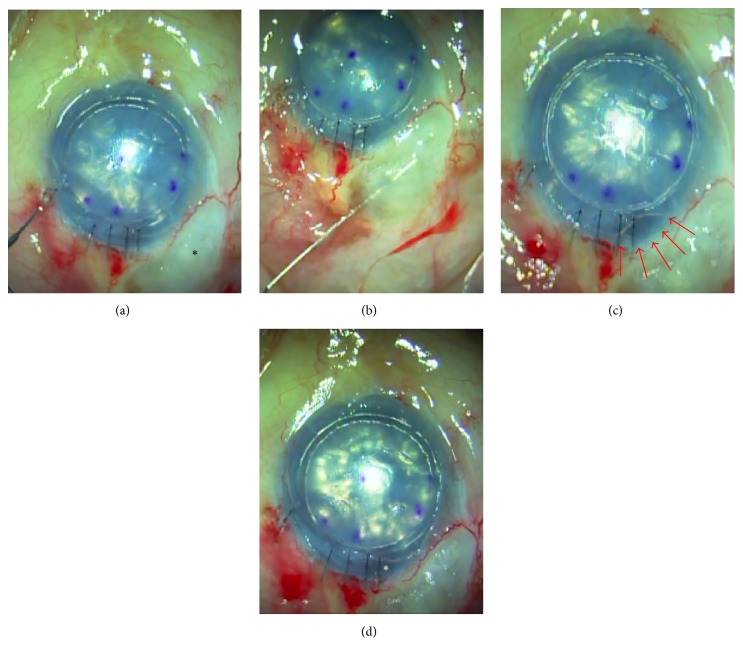
Photographs showing the modified DSAEK surgical technique using OVDs. (a) After securing the wound with interrupted 10-0 Nylon sutures, air was injected under the graft to fill the anterior chamber as much as possible. Injection was stopped when air began to migrate from the sclera flap into the bleb space (black star). (b) Then we penetrated the conjunctiva apart from the bleb by inserting a 30 G needle into the bleb and injecting Healon V. (c) Healon V was injected into the bleb until the encapsulated space was completely filled and the shape of the air bubble in the anterior chamber (AC) became deformed (arrows). (d) The DSAEK graft became firmly attached due to the pressure of the injected air, which also facilitated the draining of the remaining interface fluid. After 15 min, we confirmed that adequately high IOP was maintained by the air bubble in the AC (white star).

**Figure 2:**
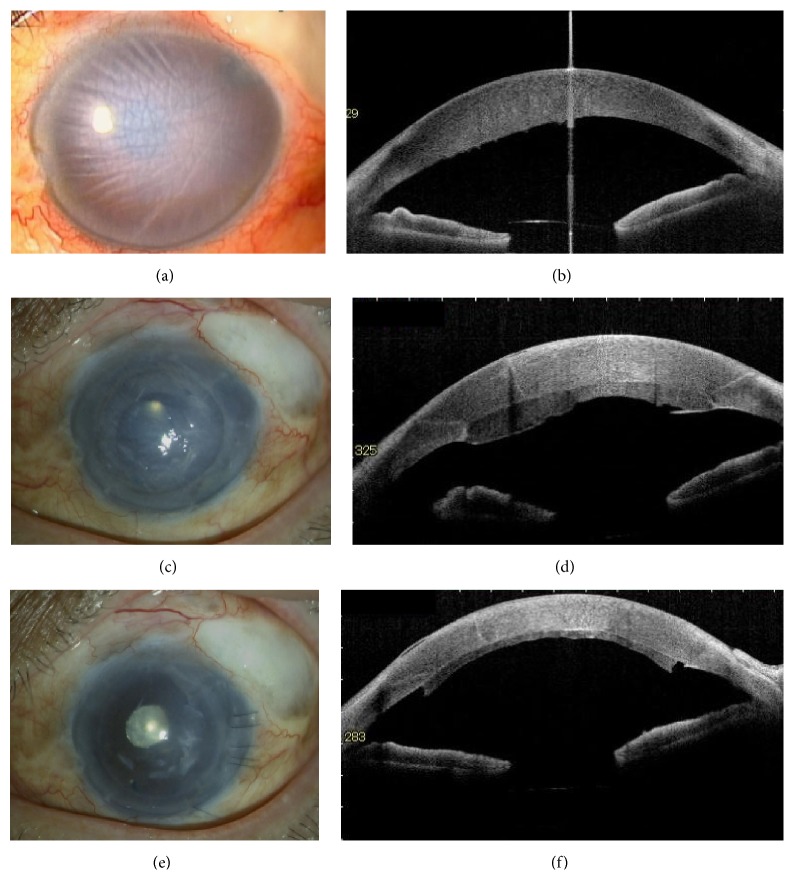
Representative slit-lamp microscopy photographs and anterior-segment optical coherent tomography images of Case 1. (a, b) Advanced bullous keratopathy with functional bleb before the secondary DSAEK surgery. (c, d) Six months after the first DSAEK surgery, the edematous DSAEK graft is observed on the back surface of the corneal stroma. The entire cornea shows remarkable edema. (e, f) One month after the secondary modified DSAEK procedure, the cornea became clear. An anterior-segment optical coherent tomography image shows that the graft was completely attached; no edema was observed.

A 67-year-old man, who had a history of cataract surgery and primary open-angle glaucoma, presented to the Department of Ophthalmology, Heart Life Hospital [Nakagusuku, Okinawa, Japan]. He had previously undergone a trabeculectomy on his left eye but developed bullous keratopathy 3 years after the surgery. The IOP (OS) after surgery remained between 6 and 8 mmHg without medication. In May 2014, he underwent the first DSAEK surgery, using the standard procedure [10]. Since this resulted in an unstable DSAEK graft attachment, repeated air rebubbling and ultimately graft sutures with 10-0 Nylon (Mani, Tochigi, Japan) were required. However, the implanted graft fell into decompensation 6 months after the surgery. The second DSAEK surgery was performed in April 2015. Briefly, corneal stab incisions were made at four points and Sheet's glide [11, 12] for intraocular lens (IOL) implantation was inserted into the anterior chamber through a 5.0 mm corneal incision [10, 13]. An anterior chamber maintainer was put through the corneal limbus. A Busin glide was used to insert the donor graft into the anterior chamber. The wound was sutured with 10-0 Nylon. Then, filtered air was injected into the anterior chamber to attach the graft to the stroma as securely as possible. The conjunctiva was penetrated, apart from the bleb, using a 30 G needle, and Healon V (AMO Japan, Tokyo, Japan) was injected into the bleb. The injection continued until the encapsulated space was completely filled and the shape of the air bubble in the anterior chamber became deformed (Figure 1). The pressure provided by this air-filling procedure pushed the remaining fluid from the interface between the graft and the back surface of the cornea, allowing it to drain through the stab incisions, which resulted in firm graft attachment. After 15 min, the air was partially replaced by artificial aqueous fluid. The IOP increased to 46–49 mmHg at 2 h after surgery. The air was partially removed, and the IOP was reduced to 28 mmHg 3 h after surgery. On the day after surgery, the IOP returned to the preoperative level of 6 mmHg, and no further elevation of IOP was observed. Optimal graft attachment was achieved without bleb leakage or microbial infection (Figure 2). The best-corrected visual acuity (BCVA) increased from 0.01 to 0.2 at 1 month, to 0.3 at 3 months, and to 0.3 at 6 months. However, he developed corneal endothelial rejection and corneal endothelial decompensation at 9 months after surgery.

**Case 2 .**

An 85-year-old man, with a history of primary-angle closure glaucoma in his right eye, who had previously undergone laser iridotomy, cataract surgery, and trabeculectomy, presented to the Department of Ophthalmology, Yokohama Minami Kyosai Hospital [Yokohama, Kanagawa, Japan]. The previously implanted IOL had become decentered, so he underwent sutured implantation of the IOL in 2012. However, he developed bullous keratopathy 1 year after the surgery. The IOP (OD) was ~5 mmHg after these surgeries. We performed our modified DSAEK, as in Case 1, in December 2014. We obtained adequate IOP during and shortly after the surgery. The IOP was 46 mmHg at 1 h and 18 mmHg at 2 h. On the day after surgery, the IOP returned to the preoperative level of 7 mmHg, and no further elevation of IOP was observed. Optimal graft attachment was achieved without bleb leakage or microbial infection. The BCVA increased from 0.01 to 0.2 at 1 month and to 0.2 at 1 year after surgery.

## 3. Discussion

In a select group of patients with soft eyes that had undergone trabeculectomy, our preliminary results indicate that judicious use of OVD into the bleb may enhance DSAEK graft adherence to the host stroma and reduce risk of postoperative graft detachment. Further evaluation with a larger number of eyes is warranted.

Several studies have reported on the outcome from DSAEK procedures in patients with prior glaucoma filtering surgeries [3–7, 14–16]. Goshe et al. [3] reported that the rate of graft dislocation in eyes with prior filtering surgery (9%) was significantly higher than that in eyes without prior filtering surgery (2%); they specifically mentioned that eyes with early postoperative hypotony of less than 7 mmHg showed significant graft detachment than eyes with normotony of more than 7 mmHg, even among eyes with blebs. They speculated that eyes with lower IOP in the early postoperative period offered less resistance to corneal deformation, allowing graft detachment with the application of minimal external force, such as simple lid pressure, squeezing, or a position change. Oster et al. [17] reported that 88% of failed DSAEK grafts had detached before failure. Those results indicated that maintaining an adequately high IOP during and shortly after surgery was essential for good graft attachment to the stroma with minimal endothelial cell damage and thus successful DSAEK outcomes.

We chose OVD as the most suitable material to inject into the bleb. Shallow and flat anterior chambers after trabeculectomy have been successfully treated via OVD injection in the anterior chamber [18–21]. The injected OVD gradually passes through the scleral flap and flows out into the bleb after a period of time; there is no occlusion of the scleral flap, and IOP is not elevated in the short period following treatment. The advantage of our method, in which the OVD is directly injected into the bleb, is the ability to reliably maintain an adequate IOP after the surgery; then, the OVD flows into the venous system, which allows the IOP to return to the normal range. When the IOP returned to the preoperative levels on the day after surgery in our cases, we believe that some of the OVD had been evacuated from the bleb during this period.

Various OVDs have been used for ophthalmic surgeries. Banitt et al. [8] compared different OVDs for the obstruction of a glaucoma drainage implant (GDI) tube and found that 1% and 1.4% sodium hyaluronate were easily purged through the tube from the anterior chamber to the subconjunctival space and that the IOP never became elevated. On the other hand, 2.3% sodium hyaluronate, with a higher viscosity and molecular weight, allowed complete filling within the anterior chamber and tube obstruction, which resulted in an increase in the IOP to 40–60 mmHg. We chose 2.3% sodium hyaluronate (Healon V) as the most suitable OVD for injection into the subconjunctival bleb in our case reports; a sufficiently high IOP was maintained for good graft attachment. The IOP returned to the preoperative levels the day after surgery. The stage of glaucoma was determined with Goldmann perimetry based on Greve's modified Aulhorn classification. The stage during the preoperative period was II, although this determination might lack accuracy due to severe corneal edema. The stage in Case 1 at 1 month after the surgery was II, as was that in Case 2, which showed less progression of glaucomatous changes. However, the results may differ with an ongoing, excessively high IOP. IOP spikes postoperatively can potentially cause progression of nerve damage with advanced glaucomatous field loss. An informed consent about potentially progression of nerve damage caused by IOP spikes should be signed before surgery and careful measurement of the intra- and postoperative IOP should be performed.

There are some difficulties with our method; first, the inserted conjunctiva that surrounds the bleb wall may induce bleb leakage, which might in turn cause serious complications, such as hypotony and a flat bleb in the early postoperative period. Bleb leakage may also induce subsequent bacterial infection. To avoid these complications, we penetrated the conjunctiva apart from the bleb, and filled the encapsulated space completely with Healon V. Another possibility is the migration of OVD between the graft and host. When the injected OVD excessively invades the anterior chamber from the bleb through the scleral flap, it may interfere with the attachment between the graft and corneal back surface. Air bubble in the anterior chamber deformed by backward flow of injected OVD indirectly indicates the existence of the invaded OVD from the bleb through the scleral flap. Therefore, we propose that injection of OVD into the bleb space should be stopped in times of the deformed air bubble in the anterior chamber. We believe that the difficulties with our method can be overcome.

## 4. Conclusions

In a select group of patients with soft eyes, judicious use of OVD into the bleb may enhance DSAEK graft adherence to the host stroma and reduce risk of postoperative graft detachment. Further evaluation with a larger number of eyes is warranted.
